# Dr. Vaughan, in Reply to Mr. Pulley, &c.

**Published:** 1799-12

**Authors:** Walter Vaughan

**Affiliations:** Rochester


### *To the Editors of the Medical and Phyfical Journal.*

GENTLEMEN,

MR. PULLEY has animadverted, with great freedom, on my communication to you of the 21ft of laft May, poffibly with the expectation of drawing from me a reply. And, although I diflike any thing like controverfy, not being deeply grounded in logic and criticifm, yet he fhall not for once be difappointed, if you will again favour me by inferting this fhort letter in your Journal.

Let me premife, gentlemen, that the hiftory of GREEN's cafe is compofed without the fmalleft mixture of hypothefis. Mr. PULLEY acknowledges befide, that, "the cafe is clearly recorded." It is plain then, that I had the truth in view, and that I did not write, like the cafe-coiners, alluded to by Dr. CULLEN, *ut Famam mihi compararem (Prolegomena. Synopfis Nofologiae Methodicae.)*

But, gentlemen, if I have fucceeded to Mr. PULLEY's mind, in the hiftorical part of my communication, I have, it feems, fallen very fhort of fuccefs in the fpeculative. This I cannot help; it is not an unufual thing for men to reafon differently, and to draw different conclufions from the fame premifes. I would not infinuate, that the profeffion of medicine is not eminently diftinguifhed by variety of knowledge and correctnefs of compofition; but I humbly imagine, that, as medical men fpeculate more than others, and as their fpeculations are more intricate, they have, confequently, more frequent opportunities of erring. Be this as it may, fuch is human nature, that, in philofophy or art, in all matters, except thofe of revelation and divine teftimony, we arrive at perfection by very flow degrees.

My conclufion, however, that GREEN's difeafe and death depended on the abforption of a poifon, accidentally introduced by the lancet of a barber, has difpleafed Mr. PULLEY; and he has been the more difpleafed, fince, by my conclufion, "the poor barber ftands convicted of having poifoned his neighbour." This then, the exculpation of "the poor barber," is, as Mr. PULLEY calls it, the "point of no fmall importance," for which my error ought to be corrected. Granted; whether for that reafon, or for any other. But, if my reafoning have been been prejudicial to the barber, I could not help it; God forbid, that I ſhould willingly do an injury to any man, in any manner.

As I could not form any notion of the origin of the ſuppoſed morbific poiſon, ſo I did not venture to gueſs at it's laws. The period between the inſertion of the ſmall-pox matter and the inflammation of the punctured part, is often leſs than three days; the period between the veneſection of Green and the inflammation was three days. The period between the introduction of ſmall-pox matter, and the ſuppuration of the punctured part, is often only four days: In Green's caſe it was only four days. So far there is ſome analogy between it and the ſmall-pox, and the *variolæ vaccinæ*: and I might trace the analogy in other reſpects; but I ſhall content myſelf, at preſent, by obſerving, that, if pain had attended the operation; if the inflammation had extended down the arm, as well as up; if there had been any induration of the venal tube; if the abſorbents had not been evidently inflamed and indurated from the puncture upwards, towards the axilla; and if the conſtitutional affection had preceded the diſeaſe of the abſorbents, I ſhould not have thought of attributing Green's ſymptoms to a morbific poiſon. Every one knows, that the abſorbent veſſels are readily inflamed by virulent matter; and none who reflects on their ſuperficial ſituation, appearance, courſe, &c. can ſuppoſe, that I miſtook for an inflammation of them, an inflammation of the vein, or of the cellular membrane. After all, what I offered was mere conjecture; and I may add, that my practice would have been preciſely the ſame, except in one particular, if I had not formed the concluſion that Green's ſymptoms proceeded from abſorption;—if ſcience alone ought to regulate practice: yet, when we cannot have ſcientific principles, we muſt content ourſelves with hypothetical ones. I never expected, that my concluſion from Green's caſe would ſupply any one with a ſingle principle of action: my concluſion is, therefore, harmleſs, if not ſtrictly correct.

I remain, Gentlemen,

Your very obedient ſervant,

*Rocheſter, Oct. 17, 1799.* WALTER VAUGHAN.

*A Caſe*

